# Clinical response trajectories and drug persistence in systemic lupus erythematosus patients on belimumab treatment: A real-life, multicentre observational study

**DOI:** 10.3389/fimmu.2022.1074044

**Published:** 2023-01-04

**Authors:** Myrto Nikoloudaki, Dionysis Nikolopoulos, Sofia Koutsoviti, Irini Flouri, Noemin Kapsala, Argyro Repa, Pelagia Katsimbri, Evangelos Theotikos, Sofia Pitsigavdaki, Katerina Pateromichelaki, Antonios Bertsias, Antonia Elezoglou, Prodromos Sidiropoulos, Antonis Fanouriakis, Dimitrios Boumpas, George Bertsias

**Affiliations:** ^1^ Rheumatology and Clinical Immunology, University Hospital of Heraklion, Heraklion, Greece; ^2^ Division of Internal Medicine, University of Crete Medical School, Heraklion, Greece; ^3^ Rheumatology and Clinical Immunology Unit, 4th Department of Internal Medicine, Attikon University Hospital, Joint Rheumatology Program, National and Kapodistrian University of Athens Medical School, Athens, Greece; ^4^ Department of Rheumatology, ‘Asklepieion’ General Hospital, Athens, Greece; ^5^ Division of Immunity, Institute of Molecular Biology and Biotechnology-Foundation for Research and Technology – Hellas (FORTH), Heraklion, Greece; ^6^ Laboratory of Autoimmunity and Inflammation, Biomedical Research Foundation of the Academy of Athens, Athens, Greece

**Keywords:** lupus, low disease activity, remission, flares, organ damage, biologics

## Abstract

**Objective:**

To obtain real-world data on outcomes of belimumab treatment and respective prognostic factors in patients with systemic lupus erythematosus (SLE).

**Methods:**

Observational study of 188 active SLE patients (median disease duration 6.2 years, two previous immunosuppressive/biological agents) treated with belimumab, who were monitored for SLEDAI-2K, Physician Global Assessment (PGA), LLDAS (lupus low disease activity state), remission (DORIS/Padua definitions), SELENA-SLEDAI Flare Index, SLICC/ACR damage index and treatment discontinuations. Group-based disease activity trajectories were modelled followed by multinomial regression for predictive variables. Drug survival was analysed by Cox-regression.

**Results:**

At 6, 12 and 24 months, LLDAS was attained by 36.2%, 36.7% and 33.5%, DORIS-remission by 12.3%, 11.6% and 17.8%, and Padua-remission by 21.3%, 17.9% and 29.0%, respectively (attrition-corrected). Trajectory analysis of activity indices classified patients into complete (25.5%), partial (42.0%) and non-responder (32.4%) groups, which were predicted by baseline PGA, inflammatory rash, leukopenia and prior use of mycophenolate. During median follow-up of 15 months, efficacy-related discontinuations occurred in 31.4% of the cohort, especially in patients with higher baseline PGA (hazard ratio [HR] 2.78 per 1-unit; 95% CI 1.32-5.85). Conversely, PGA improvement at 3 months predicted longer drug retention (HR 0.57; 95% CI 0.33-0.97). Use of hydroxychloroquine was associated with lower risk for safety-related drug discontinuation (HR 0.33; 95% CI 0.13-0.85). Although severe flares were reduced, flares were not uncommon (58.0%) and contributed to treatment stops (odds ratio [OR] 1.73 per major flare; 95% CI 1.09-2.75) and damage accrual (OR 1.83 per mild/moderate flare; 95% CI 1.15-2.93).

**Conclusions:**

In a real-life setting with predominant long-standing SLE, belimumab was effective in the majority of patients, facilitating the achievement of therapeutic targets. Monitoring PGA helps to identify patients who will likely benefit and stay on the treatment. Vigilance is required for the prevention and management of flares while on belimumab.

## 1 Introduction

Belimumab was approved for the treatment of systemic lupus erythematosus (SLE) more than a decade ago, based on the results of the BLISS-52 and -76 trials (1). The drug efficacy and safety has been further supported by additional randomized controlled trials (RCTs) capturing diverse racial patient groups, as well as their long-term extension (2–7). Recommendations issued by the European Alliance of Associations for Rheumatology (EULAR) (8), the British Society of Rheumatology (9) and other associations have placed belimumab in the management of SLE that is refractory or relapsing to standard therapies, including glucocorticoids (10). More recently, belimumab was approved for the treatment of active lupus nephritis in combination with immunosuppressive agents (11).

Post-marketing observational studies represent a valuable source of real-world evidence on the safety and effectiveness of novel therapeutic compounds and can provide useful insights to clinically pertinent topics. In an earlier practice-based study, we demonstrated good tolerability and a significant decline in disease activity in SLE patients under belimumab treatment, irrespective of the baseline serological status (12). Likewise, a number of other cohorts from different settings and regions of the world have evaluated the use of belimumab in adult SLE patients (7, 13), focusing primarily on the frequency and predictors of treatment response including disease duration, baseline disease activity, type of manifestations and pre-existing organ damage (14–18).

A complementary outcome that has been less well analysed in patients on belimumab (19) is drug retention, which is reflective of multiple parameters (efficacy, including longevity of treatment response, safety, as well as patient and physician preferences) (20). This becomes relevant in view of the variable rates of patients who discontinue treatment in the above-mentioned studies. To this end, identifying the best candidates for belimumab treatment represents an important unmet need. This is further perplexed by the gradual mode of drug action, with responders accumulating over a period of 6-12 months (12, 15, 17).

Herein, we report on the extended follow-up of our initial belimumab cohort from three hospital centres, enriched with additional cases to reach a total 188 patients monitored in real-life setting. In addition to the effects on disease activity, use of glucocorticoids and achievement of the low disease activity and remission goals, we analyse our dataset for longitudinal trajectories of clinical response to belimumab and identify relevant predictors. Also, we assess the retention of belimumab in our study population and determine factors linked to efficacy- and safety-related drug discontinuation. Finally, we examine the incidence of flares and organ damage as well as their prognostic impact in patients under belimumab treatment.

## 2 Materials and methods

### 2.1 Setting and patients

This is a prospective observational study of SLE patients enrolled by consecutive sampling between 12/2014 and 08/2021 from three centres in Greece. Inclusion criteria were: a) age ≥18 years at inclusion, b) fulfilment of the 1997 American College of Rheumatology (ACR) revised criteria (21) or the 2012 Systemic Lupus International Collaborating Clinics (SLICC) criteria (22) for SLE classification, c) active disease despite conventional, standard-of-care treatment, according to physician judgment and ascertained with a PGA ≥1 (scale 0 to 3) (23) and SLE disease activity index (SLEDAI)-2K >0 (24), d) treatment with belimumab (approved intravenous or subcutaneous formulation) for at least 3 months. Patients gave informed consent and the study was approved by the Ethics Committees. From an initial 189 eligible patients, one was excluded due to incomplete clinical data. Forty-three patients were included in our earlier study (12) but herein, their extended follow-up was analysed.

### 2.2 Monitoring of disease activity and other clinical parameters

The collaborating centres use homogenised, structured forms for SLE assessment (25, 26). Patients were evaluated quarterly up to 18 months and every 4-6 months thereafter. Due to the COVID-19 pandemic or patient-related reasons, some evaluations were not carried out at the predetermined time-point, thus reducing the valid sample size by *n*=8-15. The following variables were monitored (27): demographics (gender, nationality, date of birth); date of diagnosis and fulfilment of the SLE classification criteria; smoking status (never, former, current smoker); major comorbidities; previous and concomitant treatments and their dosage (including glucocorticoids, immunomodulatory/immunosuppressive or biologic agents); disease activity [SLEDAI-2K (24), PGA (23)]; flares (SELENA-SLEDAI Flare Index (28) modified to include mycophenolate and rituximab under the definition of *severe flare*); organ damage [SLICC/ACR Damage Index (29)]; and adverse events including death (MedDRA recording system; https://www.meddra.org/faq/meddra-general). In accordance to the standard practice of the participating clinical canters, PGA was scored before immunological tests were available. Lupus Low Disease Activity State (LLDAS) (30) and clinical remission [defined according to DORIS (31) and Zen et al. (32, 33)] were assessed. Belimumab discontinuations (permanent or interruption of treatment for more than 3 months) were captured as due to: a) inefficacy/unsatisfactory response; b) safety/adverse events; c) pregnancy (or wish for); d) other reasons. Following pseudo-anonymization, data were entered into a secure electronic registry (27).

### 2.3 Statistical analysis and group-based trajectory modelling

Median values with interquartile ranges (IQR) were calculated for continuous variables. Between-groups comparisons were performed with the chi-squared and Kruskal-Wallis tests. Longitudinal trends in activity indices were analysed with the non-parametric Skillings-Mack test. To account for treatment attrition, Lundex correction was applied (34). Clusters of patients following similar patterns of disease activity over time were identified by GBTM using the STATA “*traj*” plugin (35). SLEDAI-2K, PGA and time polynomials (0 to 24 months) were used as covariates and the optimal number of clusters and best-fitting model were determined according to the lowest adjusted Bayesian information Criterion value. The criteria by Strauss et al. (36) were considered with a minimum 10% of patients included in the smallest cluster. Discovered clusters were combined into groups of clinical response to belimumab, which were then compared for clinical characteristics using the chi-squared test and multinomial regression. Kaplan-Meier survival curves were used to estimate efficacy- and safety-related drug retention (treating unrelated causes of discontinuations as censored observations). Univariable and multivariable-adjusted Cox proportional hazards regression was performed to determine risk factors for drug discontinuation. The association between flares and outcomes was examined by logistic regression. All analyses were performed using STATA (version 16) and SPSS (version 25). Two-tailed p-values <0.05 were considered statistically significant.

## 3 Results

### 3.1 Belimumab reduces disease activity and glucocorticoid dose in patients with long-standing SLE

We analysed 188 SLE patients with median (IQR) age and disease duration of 48.4 (19.5) and 6.2 (9.5) years, respectively (**Table 1**). The vast majority (95.7%) had been previously treated with immunosuppressive or biologic agents (median [IQR]: 2 [2] drugs) including cyclophosphamide (*n*=33) and rituximab (*n*=20). Organ damage was present in 39.4% of patients. At inclusion, most frequent SLEDAI-2K domains were arthritis (86.7%), rash (56.4%), immunology (37.2%), hair loss (33.5%) and mucosal ulcers (23.4%).

**Table 1:** Baseline demographic and clinical characteristics of SLE patients treated with belimumab.

	N (%) or median (IQR)^1^
Gender (female)	180 (95.7)
Age (years)	48.4 (19.5)
Disease duration (years)	6.2 (9.5)
Non smokers	91 (48.4) ^1^
Previous treatments (excluding glucocorticoids)	
Hydroxychloroquine	148 (78.7)
Methotrexate	137 (72.9)
Leflunomide	34 (18.1)
Calcineurin inhibitors	16 (8.5)
Azathioprine	102 (54.3)
Mycophenolate	26 (13.8)
Thalidomide	4 (2.1)
Cyclophosphamide	33 (17.6)
Rituximab	20 (10.6)
IVIG	7 (3.7)
Other biologics	4 (2.1)
Disease state	
SLEDAI-2K	8 (3)
SLEDAI-2K ≥10	43 (22.9)
Active serology ^2^	70 (37.2)
PGA	1.5 (0.5)
Organ damage (SDI >0)	74 (39.4)

^1^ Interquartile range; ^2^ Data available on n=174 patients; ^3^ low C3/C4 and/or high anti-dsDNA titres.

**Figure 1:**
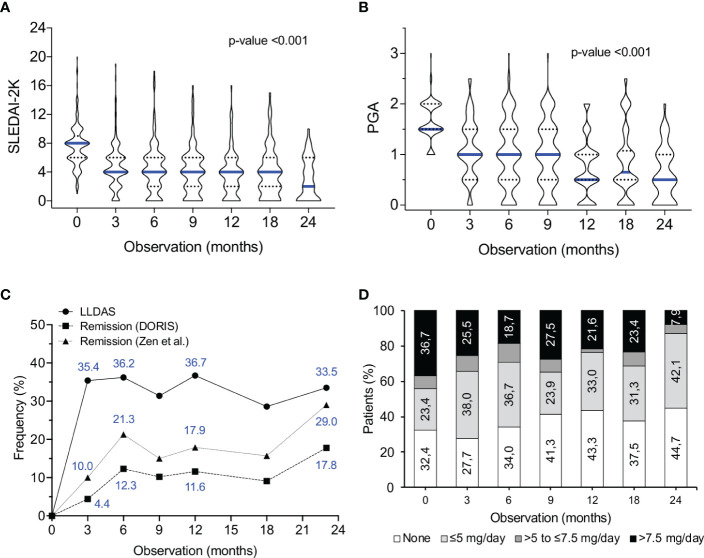
Effect of belimumab treatment on SLE disease activity, attainment of therapeutic goals and dose of glucocorticoids. **(A, B)** Violin plots of SLEDAI-2K **(A)** and PGA **(B)** values at consecutive time-points during belimumab treatment. Blue lines represent median values. The non-parametric Skillings-Mack test was used to determine the statistical significance of longitudinal trends. The number of analysed patients were 178, 164, 136, 109, 74 and 48 at the 3, 6, 9, 12, 18 and 24-month time points, respectively. The number of patients with shorter follow-up were 0, 6, 16, 21, 40 and 53, respectively. **(C)** Rates (Lundex-corrected) of attainment of low disease activity (LLDAS (30)), remission (according to the DORIS (31) and the Zen et al. (32, 33) definitions) in belimumab-treated patients. **(D)** Stacked bars demonstrating the glucocorticoid dosage level (none, >0 and ≤5 mg/day, >5 to ≤7.5 mg/day, >7.5 mg/day prednisone equivalent) in patients under treatment with belimumab.

Under belimumab treatment, a significant decrease in disease activity was observed, starting as early as 3 months (**Figures 1A, B** **)**. Attainment of low disease activity (LLDAS; Lundex-corrected) was 35.4%, 36.7% and 33.5% at months 3, 12 and 24, respectively (**Figure 1C**). In terms of remission, 12- and 24-month rates were of 11.6% and 17.8% (DORIS definition (31)), and 17.9% and 29.0% (Padua definition (33)), respectively. Patients who were off-glucocorticoids increased from 32.4% at baseline to 43.3% (month 12), paralleled by a decrease in those receiving >7.5 mg/day prednisone equivalent (from 36.7% to 21.6%) (**Figure 1D**). Collectively, these results indicate that in a significant proportion of patients with active long-standing SLE, belimumab helps to achieve the established treatment goals by reducing disease activity and use of glucocorticoids.

### 3.2 Distinct trajectories of disease activity and response to treatment with belimumab

**Figure 2:**
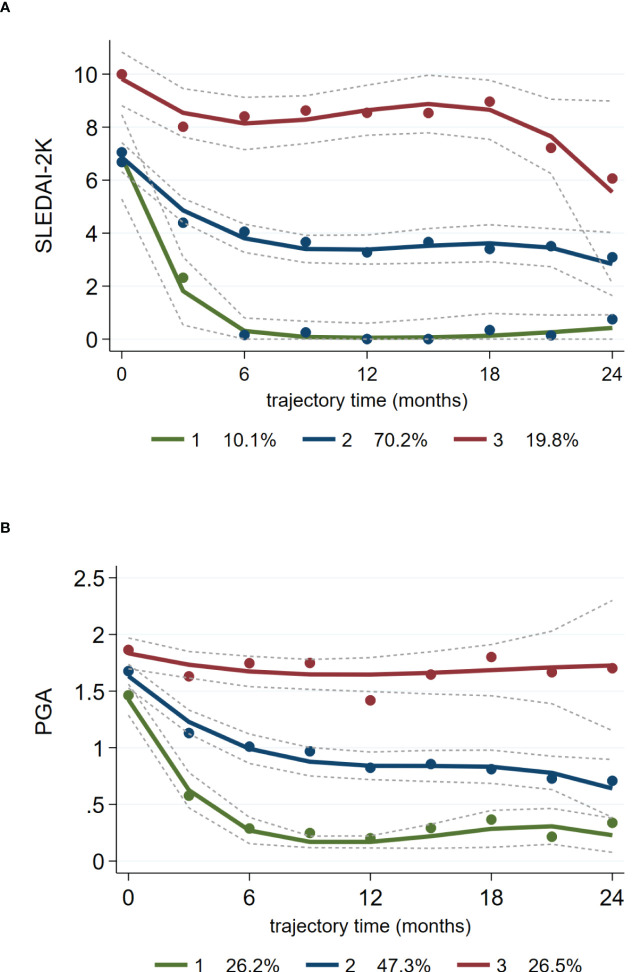
Distinct trajectories of disease activity (SLEDAI-2K, PGA) in SLE patients treated with belimumab. Group-based trajectory modelling of SLEDAI-2K **(A)** and PGA **(B)** during belimumab treatment was performed as described in the Methods section. Solid lines (coloured) represent the parameter estimates of each model, and dashed lines represent the 95% confidence interval of the estimates. Dots are calculated from the actual data where each individual’s responses are weighted based on the posterior probabilities of group membership. Three distinct trajectories of SLEDAI-2K and PGA are indicated with different colours (top-red, middle-blue, bottom-green). The percentages at the bottom of each panel correspond to the proportion of SLE patients belonging to each trajectory.

The summary-level data shown in **Figures 1A, B** were suggestive of inter-individual variation regarding longitudinal changes in activity indices during belimumab treatment. We therefore examined whether patients can be grouped into homogenous trails of disease activity. GBTM identified three distinct trajectories of SLEDAI-2K and PGA (extending to the 24-month time-point) (**Figures 2A, B** **)**. The most prevalent trajectory (70.2% according to SLEDAI-2K, 47.3% according to PGA) included patients who demonstrated a gradual response to low disease activity levels (Traj_2: *blue-coloured line*). A second trajectory (10.1% according to SLEDAI-2K, 26.2% according to PGA) was characterised by rapid, significant reduction of activity to minimal or remission levels (Traj_1: *green-coloured line*), whereas a third one (19.8% according to SLEDAI-2K, 26.5% according to PGA) corresponded to patients with non-improving, persistent activity (Traj_3: *red-coloured line*).

By combining the SLEDAI- and PGA-derived clusters, we categorised patients into three groups (according to the trajectories they belonged) designated as *complete responders* (CR: 25.5% of the cohort; Traj_1 in both indices), *partial responders* (PR: 42.0; Traj_2 in at least one index, but not Traj_3 in any index) and *non-responders* (NR: 32.4%; Traj_3 in at least one index). To verify our method, we assessed the SLEDAI-2K and PGA levels, as well as the rates of LLDAS and remission at consecutive time-points across the three groups (**Supplementary Table S1**). We observed consistent group-based differences in the aforementioned indices, signifying worse outcome in the NR *versus* PR *versus* CR group. Similar results were obtained when GBTM was performed using disease activity data (SLEDAI-2K, PGA) of the first 12 months (**Supplementary Figure S1** and **Table S2**).

### 3.3 Baseline factors are associated with clinical response trajectories in belimumab-treated SLE patients

**Figure 3:**
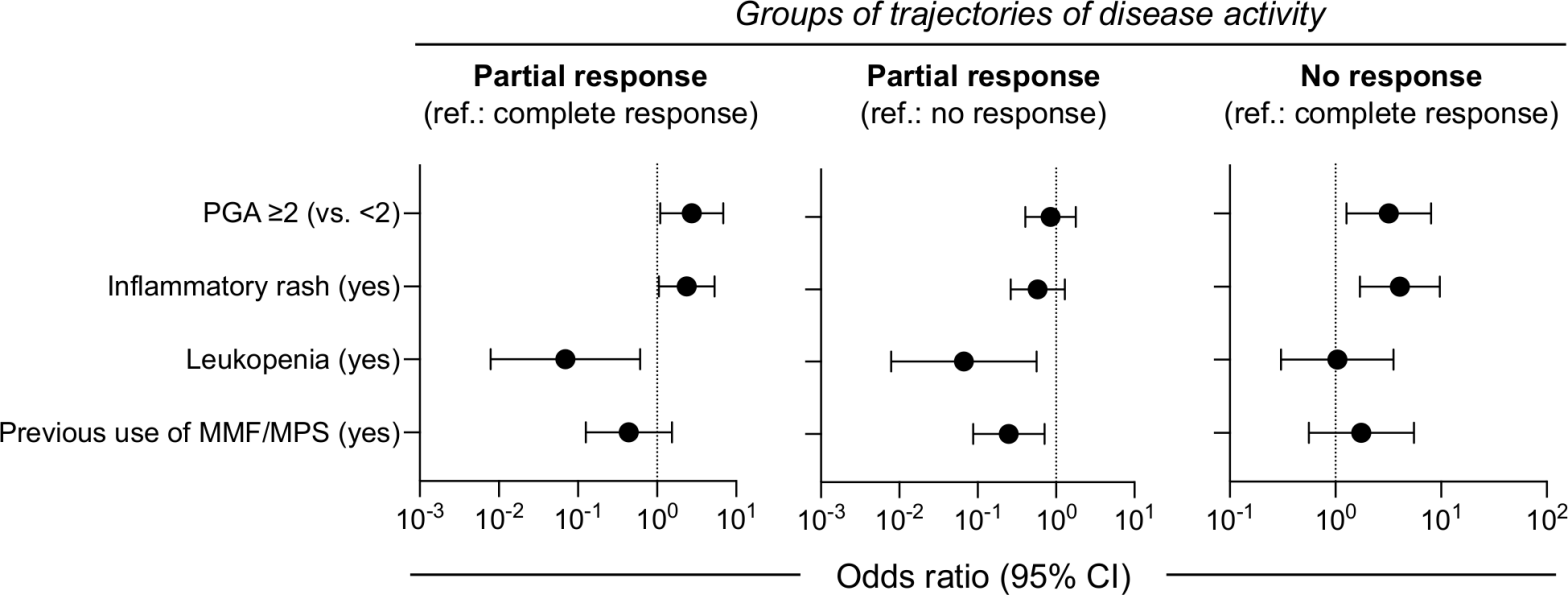
Baseline factors may predict clinical response trajectories in belimumab-treated SLE patients. Multinomial regression analysis was performed to identify baseline clinical parameters associated with the trajectory-based groups of response to belimumab. Patients with baseline PGA ≥2.0 or inflammatory rash were more likely to belong to the partial response (PR) (odds ratio [OR] 2.74; 95% confidence interval [95% CI] 1.09–6.85 and OR 2.37; 95% CI 1.05–5.35, respectively) and the non-response (NR) (OR 3.19; 95% CI 1.27–8.06 and OR 4.07; 95% CI 1.70–9.71, respectively) than the complete response (CR) group. Leukopenia was linked to reduced risk for PR as compared to CR (OR 0.07; 95% CI 0.01–0.61) and NR (OR 0.07; 95% CI 0.01–0.56). Prior use of mycophenolate was associated with lower risk for PR versus NR (OR 0.25; 95% CI 0.09–0.71).

Predicting response to belimumab over time rather at a single time-point, can be particularly helpful in clinical decision process. We screened for demographic and clinical variables associated with the three trajectory groups by performing first, univariable analysis (**Supplementary Table S3**) followed by adjusted multinomial regression. Patients with baseline PGA ≥2.0 or inflammatory rash were more likely to belong to the PR and the NR than the CR group (**Figure 3**; *exact estimates provided in the figure legend*). Presence of leukopenia was linked to reduced risk for PR as compared to CR and NR. Finally, prior use of mycophenolate was associated with lower risk for PR versus NR. In the absence of high baseline PGA (≥2.0) and rash, the probability for NR to belimumab was low (14%). Patients with these aforementioned risk factors had intermediate risk for NR (41%), which was increased further in the presence of leukopenia or earlier use of mycophenolate (62%). Pending confirmation in additional cohorts, these results might be useful for individualized predictions of belimumab response trajectories.

### 3.4 Early improvement in PGA and use of hydroxychloroquine predicts increased retention of belimumab treatment

**Figure 4:**
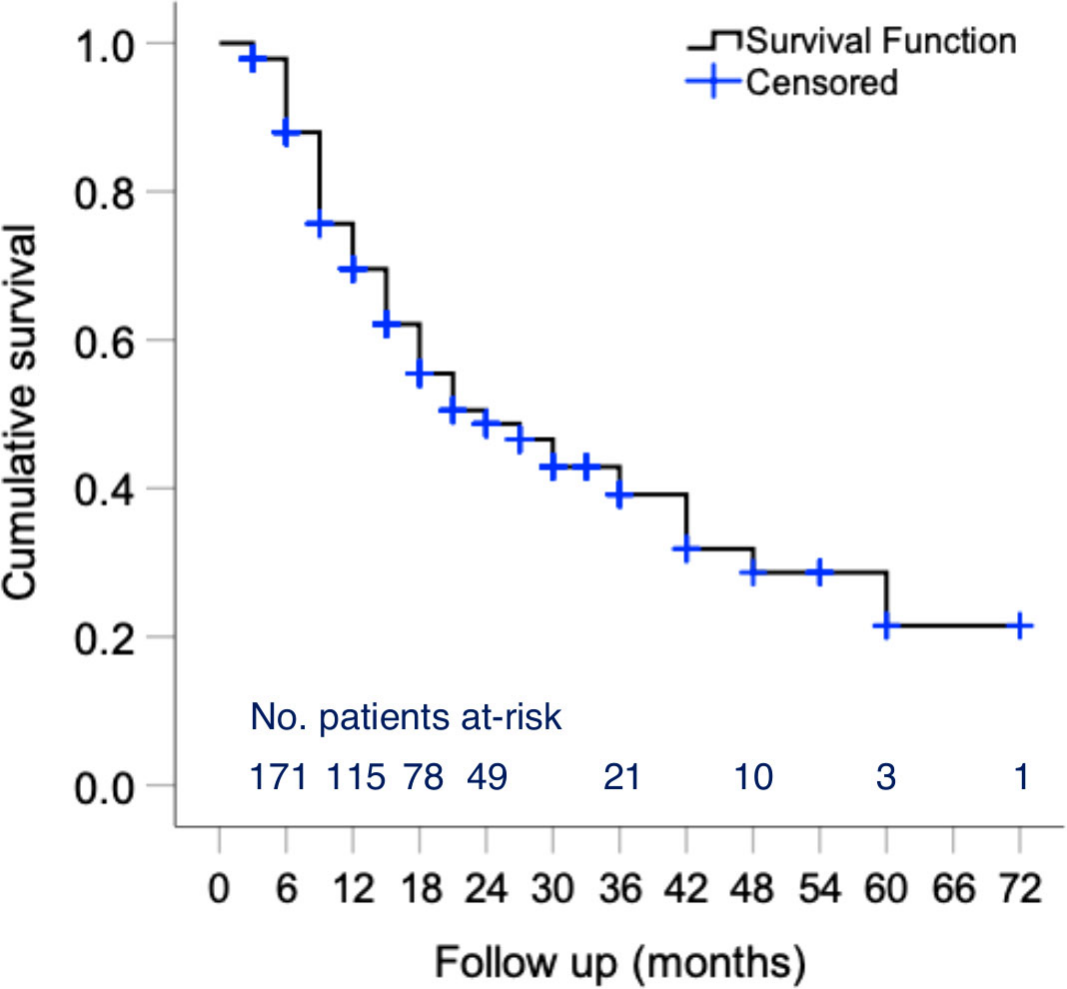
Persistence of belimumab treatment in patients with active SLE. Kaplan–Meier survival curves of belimumab in active SLE patients. The number of individuals at-risk entering each time interval is shown at the bottom of the plots. One- and two-year efficacy-related retention rates were estimated at 71% and 61%, respectively. Safety-related retention rates at 1 and 2 years were estimated at 93% and 85%, respectively.

In addition to changes in disease activity, drug persistence is a useful outcome measure for the benefit/risk evaluation of novel therapeutic agents. During a median (IQR) follow-up of 15 (15) months, 93 patients discontinued belimumab (**Figure 4**) primarily due to unsatisfactory response (physician judgment) (*n*=59; 31.4% of total cohort), adverse events (*n*=19; 10.1%) or patient decision (including wish for pregnancy) (*n*=9; 4.8%). Estimated 1- and 2-year efficacy-related retention rates were 71% and 61%, respectively. Notably, drug survival plots showed clear separation between the three clinical response trajectory groups (**Supplementary Figure S2**), further corroborating the clinical relevance of our cluster analysis. Safety-related retention rates at 1 and 2 years were 93% and 85%, respectively. Most frequent events leading to treatment cessation were infections (*n*=7), psychiatric events (*n*=4) and malignancies (*n*=3) (**Supplementary Table S4**).

Cox regression was performed to determine factors associated with belimumab discontinuation. First, we analysed efficacy-associated stops and confirmed – by univariate analysis – the prognostic impact of baseline SLEDAI-2K and PGA, whereas inflammatory rash (p=0.053) and concomitant use of immunosuppressives (p=0.079) did not reach statistical significance (**Supplementary Table S5**). We next examined whether early trends in disease activity might predict drug persistence. Improvement in PGA (by ≥0.5-units) at 3 months correlated with significantly lower risk for belimumab cessation, contrary to reduction in SLEDAI-2K (by ≥4 units) or normalization of serology that did not. Similarly, we found no association with other factors such as disease duration, tobacco use, previous immunosuppressive treatments or the use of glucocorticoids (*data not shown*). In the multivariable-adjusted model, baseline PGA (HR 2.78 per 1-unit; 95% CI 1.32-5.85) and early PGA improvement (HR 0.57; 95% CI 0.33-0.97) were retained as predictors of efficacy-related retention of belimumab. With regards to safety-related survival, only baseline use of hydroxychloroquine was found to be protective against belimumab discontinuation (HR 0.33; 95% CI 0.13-0.85). No deaths were noted.

### 3.5 Flares under belimumab treatment carry an increased risk for drug cessation and damage accrual

Belimumab has been shown to reduce the risk for lupus flares, which nevertheless, can occur especially during the early phases of treatment (6, 37). In our cohort, 43.4% (*n*=82) of patients developed an average 1.45 mild/moderate flares, and 21.3% (*n*=40) developed an average 1.40 severe flares, according to the SFI definitions. Accordingly, the incidence rate for mild/moderate and severe flares was 40.9 and 19.2 per 100 patient-years, respectively (**Supplementary Figure S3**). Severe flares tended to become less frequent with prolongation of belimumab treatment, with incidence rates of 20.7, 18.3 and 6.8 per 100 patient-years during the 0-12, 12-24 and 24-36-month intervals, respectively.

We explored the impact of flares on pertinent outcomes, including treatment withdrawal and organ damage accrual. We found that 35 of 59 efficacy-related belimumab discontinuations occurred in the context of concurrent or preceding (3 months) flares (19 severe, 16 mild/moderate), whereas the remaining 24 stops were due to stably active (non-improving) disease. Accordingly, each severe flare was associated with increased risk (OR 1.73; 95% CI 1.09-2.75) for belimumab discontinuation. Since not all severe flares led to treatment cessation, we examined whether this risk is affected by changes (e.g., initiation, dose increase or switches) in background immunosuppressive therapy (excluding glucocorticoids). Out of 40 SLE patients who experienced a severe flare, belimumab was stopped in 5/10 patients who changed therapy *versus* in 17/30 who did not (p=ns).

During follow-up, 20 patients (10.7%) accrued new damage and this risk was elevated in patients with mild/moderate flare(s) (OR 1.83 per flare; 95% CI 1.15-2.93). Likewise, patients who developed damage had almost twice as many flares than those who did not (mean ± standard deviation: 1.10 ± 0.97 versus 0.56 ± 0.81, p=0.006). A similar trend was observed also for severe flares, although it did not reach statistical significance probably due to the small sample size. Altogether, these findings highlight a significant burden of flares during belimumab treatment including an increased risk for drug discontinuation.

### 3.6 Clinical outcomes in belimumab-treated patients who had previously received cyclophosphamide or rituximab

A total 40 patients (21.3%) had been previously treated with cyclophosphamide or rituximab, which are generally used in severe or refractory SLE (8). A separate analysis in these patients revealed clinical outcomes that were comparable to whole study population of belimumab-treated patients. Specifically, attainment of LLDAS (Lundex-corrected) at 3, 12 and 24 months were 34.2%, 37.5% and 33.0%, respectively. The corresponding rates of DORIS remission (31) were 10.2%, 7.9% and 19.8%, and of Padua remission (33) were 17.4%, 14.2% and 24.8%, respectively. In terms of trajectory-defined clinical response, 12 patients (30.0%) had CR and the remaining 28 patients were assigned evenly to the PR and NR groups (n=14; 35%.0 in each). Mild/moderate and severe flares developed in 14 and 6 patients, corresponding to incidence rates of 37.7 and 12.6 per 100 patient-months, respectively. During follow-up, 4 patients (10.0%) accrued new damage and 19 patients (47.5%) discontinued treatment (n=11 due to unsatisfactory response, n=3 due to adverse events).

## 4 Discussion

We present the results from a multicentre, real-life study in active SLE patients treated with belimumab. Our cohort, enriched with long-standing, difficult-to-treat lupus, showed a decline in disease activity and use of glucocorticoids, accompanied by an important proportion of patients who reached low disease activity or remission. Trajectory analysis identified three distinct groups of clinical response to belimumab, with high baseline activity and inflammatory rash denoting patients at moderate-to-high risk for non-response. On the other hand, improvement in PGA at 3 months predicted longer treatment persistence. Finally, we found that lupus flares were not uncommon and burdened the disease course of patients under belimumab therapy resulting in drug discontinuation and organ damage accrual.

Our findings corroborate evidence from earlier cohorts demonstrating significant improvement in disease activity indices, such as the SLEDAI and PGA during belimumab treatment (12–15, 19, 38, 39). Fewer data are available on the attainment of low disease activity and remission (12, 15, 17, 18), which are recommended therapeutic goals to improve disease and patient outcomes (8, 40). Although direct comparisons are hampered by differences in patient characteristics and outcome definitions, rates of remission [according to Zen et al. (32, 33)] were similar between our and the Italian BeRLiSS study at 6 and 12 months (21.3% *versus* 23.3% and 17.9% *versus* 23.3%, respectively) (15). In line with previous reports (12, 17, 38), LLDAS was also achieved by a considerable proportion of our cohort. Notably, our study comprised patients with predominant long-standing (74.5% with duration longer than two years) or recalcitrant disease (29.3% with prior use of more than two immunosuppressants/biologics), which further supports the use of belimumab to control activity and help accomplish the recommended treat-to-target goals.

Previously, observational studies have ascertained clinical response to belimumab by the SRI-4 (15, 17, 19, 38), a composite outcome measure developed in the context of RCTs (41). Herein, we performed group-based trajectory analysis for the unsupervised discovery of patient clusters with similar longitudinal behaviours of disease measures (42). By modelling both SLEDAI-2K and PGA as complementary indices of SLE activity/severity (43), we identified and verified three patterns of response to belimumab. About one-fourth of patients improved to absent or minimal disease activity (*complete responders*), another 42% showed clinically-relevant improvement to low disease activity levels (*partial responders*), whereas one-third of patients had no or minimal decline in activity (*non-responders*). The latter percentage is somewhat higher than the frequency of SRI-4 non-responders reported in the above-mentioned cohort studies (ranging 18.3-30.3% at months 12-24) (15, 17, 38). Our data provide real-life estimates of complete, partial and no response to belimumab which conforms to physicians’ impression over the drug effectiveness in tertiary or referral clinics (14, 44, 45).

The identification of patient subsets who are more likely to benefit from belimumab remains an unmet clinical need. By comparing our trajectory-based groups, we found that high baseline activity (PGA ≥2) and inflammatory rash were negative predictors for CR. Of note, leukopenia and prior use of mycophenolate increased the likelihood for NR as compared to PR to belimumab. These findings reiterate those by Gatto et al. (15) and Sbeih et al. (17) who identified skin/mucocutaneous involvement as a predictor for lower or delayed attainment of remission or low disease activity. The former study also found high baseline activity (SLEDAI-2K ≥10) to predict less time spent on remission or low disease activity states (15). Nevertheless, in our study, the majority of patients with baseline PGA ≥2 or rash were belimumab responders (57.0% and 59.5%, respectively), suggesting these patient groups can still be considered candidates for the treatment.

Contrary to other reports (15, 17, 38), we observed no significant associations of response to belimumab with baseline organ damage, use of tobacco and disease duration, which might be due to the specific characteristics of our cohort. Nonetheless, a strategy for timely initiation of disease-modifying treatment such as belimumab, before accumulation of organ damage, as well as for abstinence from tobacco use, cannot be over-emphasized in SLE patients (8). The relationship of leukopenia with differential response to belimumab is interesting although it might be partially confounded by other disease features (e.g., patients with leukopenia had increased frequency of vasculitis and reduced frequency of rash and arthritis; data not shown). From a clinical standpoint, the above-mentioned predictors can be useful to determine the *a priori* risk for belimumab failure, therefore facilitating individualized monitoring and treatment, although further validation will be required.

In line with our trajectory analysis, about 31% of SLE patients discontinued belimumab due to unsatisfactory efficacy. This percentage lies within the range of previously reported efficacy-related drug withdrawal rates (16-45%) (12, 15–17, 19, 38, 46, 47). Most withdrawals occurred at 6-12 months after treatment initiation, implying that physicians consider this time frame as most relevant to adjudicate belimumab effectiveness. Importantly, Cox-regression revealed that independent of baseline activity, reduction in PGA at 3 months predicted increased drug retention. These data support the use of PGA as a simple instrument to measure disease activity/severity during belimumab treatment and also, suggest that patients without an early (at 3 months) improvement should be monitored closely for possible treatment adjustments. An important observation was the increased safety-related belimumab retention associated with the use of hydroxychloroquine, which adds to its multifaceted beneficial effects in SLE (48). Hypothetical explanations might include the presumed effect of hydroxychloroquine on reducing the risk for infections and/or their severity (49), or inhibition of the formation of anti-drug antibodies associated with adverse events, similar to what has been described for other conventional synthetic disease modifying antirheumatic drugs (50). Of note, patients who did not use hydroxychloroquine had increased age as compared to their counterparts who used hydroxychloroquine (median [IQR]: 51.4 [15.1] versus 46.6 [18.2] years, p=0.005). However, age did not influence the risk for safety-associated belimumab discontinuation (**Supplementary Table S5**), although some confounding effect cannot be entirely excluded.

Similar to the BeRLiSS study (15), lupus exacerbations were not uncommon during belimumab treatment although severe flares tended to decrease over time. We confirmed the prognostic impact of flares, in particular their association with increased risk for new organ damage and belimumab discontinuation. Although our sample size precluded robust analyses, we found no evidence that these risks were mitigated by modifications in background immunosuppressive therapy. These findings emphasise the need to consider flare prevention strategies even under treatment with belimumab; examples include the gradual, rather than abrupt, tapering and withdrawal of glucocorticoids (51) and regular evaluation of patient compliance to prescribed therapy.

Our study limitations include its practice-based design with lack of a comparator arm and some missing assessments thus, reducing the number of valid observations (by *n*=8-15 at each time-point). Consequently, the sample size may be considered insufficient to detect modest statistical associations. To account for treatment attrition, our results were based on Lundex correction, GBTM that handles missing data by fitting the model using maximum likelihood estimation (35), and time-to-event Cox-regression analysis. The reduced number of analysed patients (especially after month 18) affects the certainty of the results as indicated by the widening of the confidence intervals of the corresponding trajectory plots. Nonetheless, our sensitivity analysis with 12-month-based GBTM yielded similar response groups. Moreover, since PGA represents the physician’s perspective on patient disease activity and the decision to maintain treatment was made at the physician discretion, a possible confounding bias cannot be excluded, although we observed a relationship between trends in PGA with respective changes in objective measures of disease activity. On the other hand, our study is one the largest observational cohorts with homogenous data collection providing real-world efficacy and safety evidence.

Conclusively, in an unselected SLE cohort, belimumab was shown to reduce disease activity and glucocorticoid usage, thus facilitating the implementation of the treat-to-target strategy even in patients with long-standing, difficult-to-treat disease. Standard baseline parameters, such as the PGA, rash, leukopenia and prior use of mycophenolate can be combined to forecast clinical response to belimumab and potentially enable personalized monitoring strategies. We propose the use of PGA for monitoring early changes in disease activity associated with long-term retention of belimumab. During treatment, vigilance is required for the prevention, early identification and management of flares.

## Data availability statement

The original contributions presented in the study are included in the article/**Supplementary Material**. Further inquiries can be directed to the corresponding author.

## Ethics statement

The studies involving human participants were reviewed and approved by University Hospital of Heraklion IRB (14021/17-02-2021). The patients/participants provided their written informed consent to participate in this study.

## Author contributions

MN performed data extraction and entry. DN, SK, NK, SP, KP and ET evaluated patients and performed data extraction from medical charts and forms. IF assisted in the developed of the registry and curated the data. AR, PK, AE and PS evaluated patients receiving belimumab. AB assisted in statistical analysis and trajectories modelling. AF and DB evaluated patients receiving belimumab, edited the manuscript and co-supervised the study. GB supervised the study, performed statistical analysis and drafted the manuscript. All authors contributed to the article and approved the submitted version.
