# Supplementary figures and images for: Clinical response trajectories and drug persistence in systemic lupus erythematosus patients on belimumab treatment: A real-life, multicentre observational study
Source: Front Immunol. 2023 Jan 4;13:1074044. doi: 10.3389/fimmu.2022.1074044 (PMC9845912; doi:10.3389/fimmu.2022.1074044)

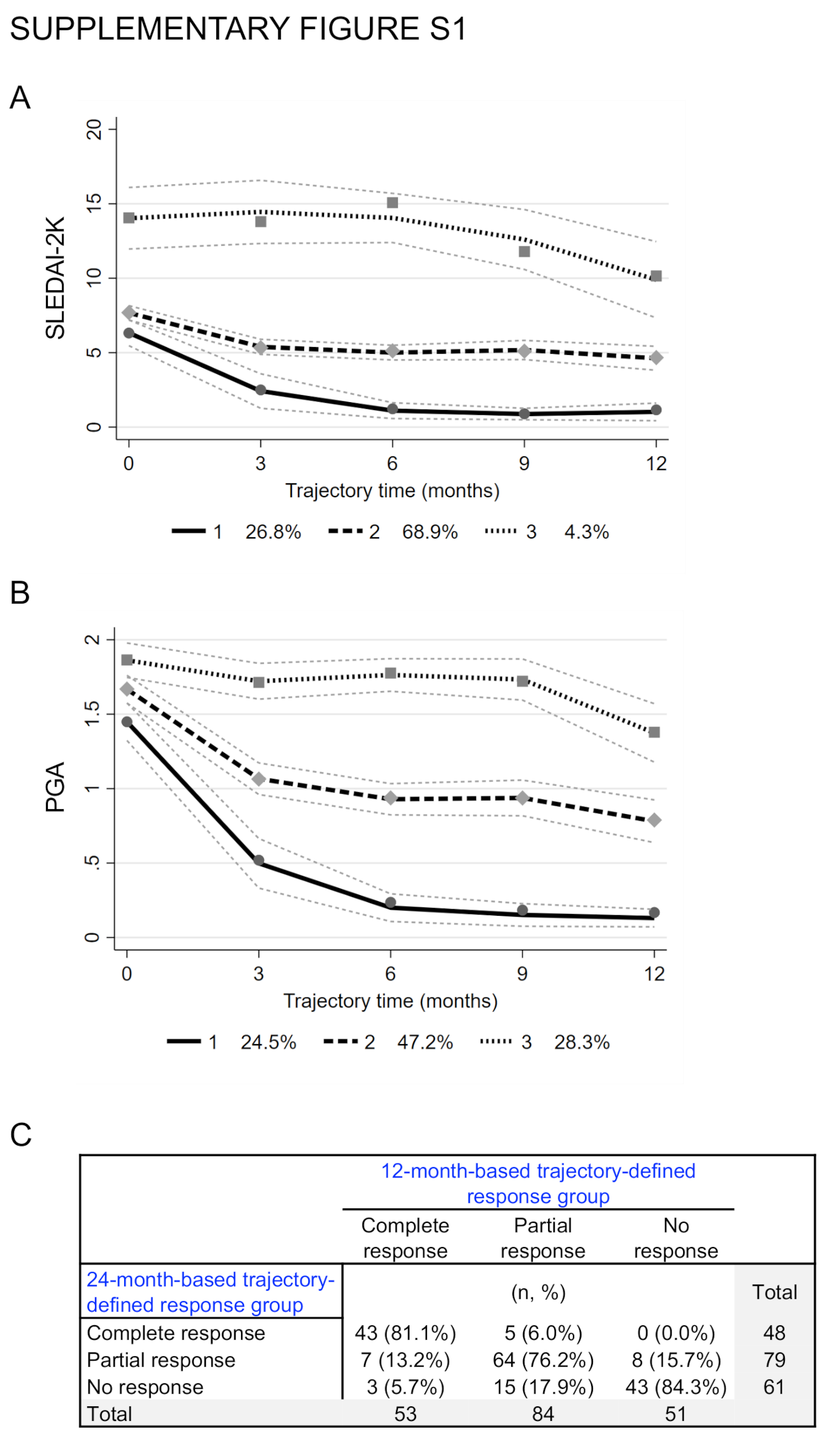

Supplement: Supplementary file 1 [file Image_1.tif]

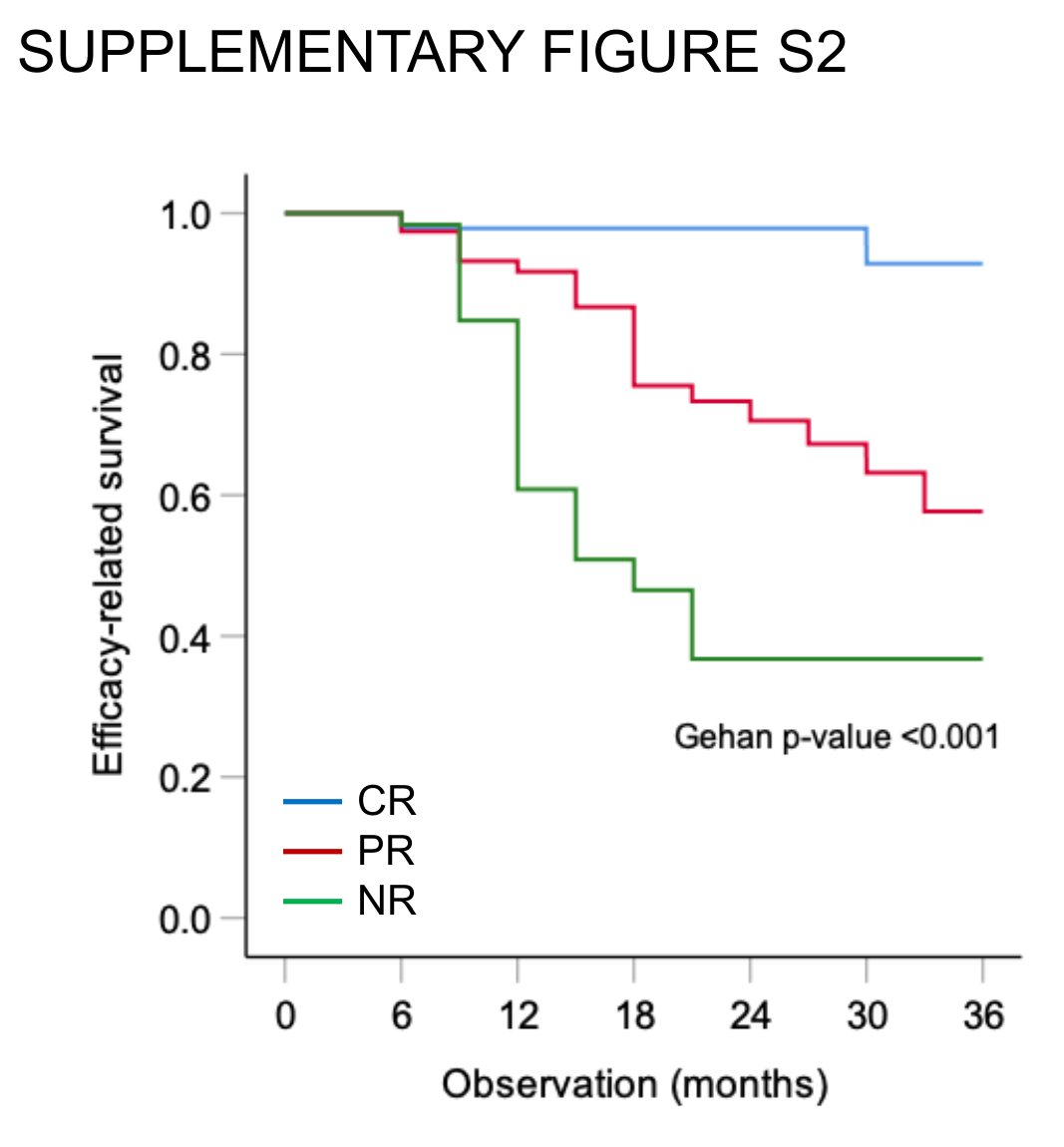

Supplement: Supplementary file 2 [file Image_2.tif]

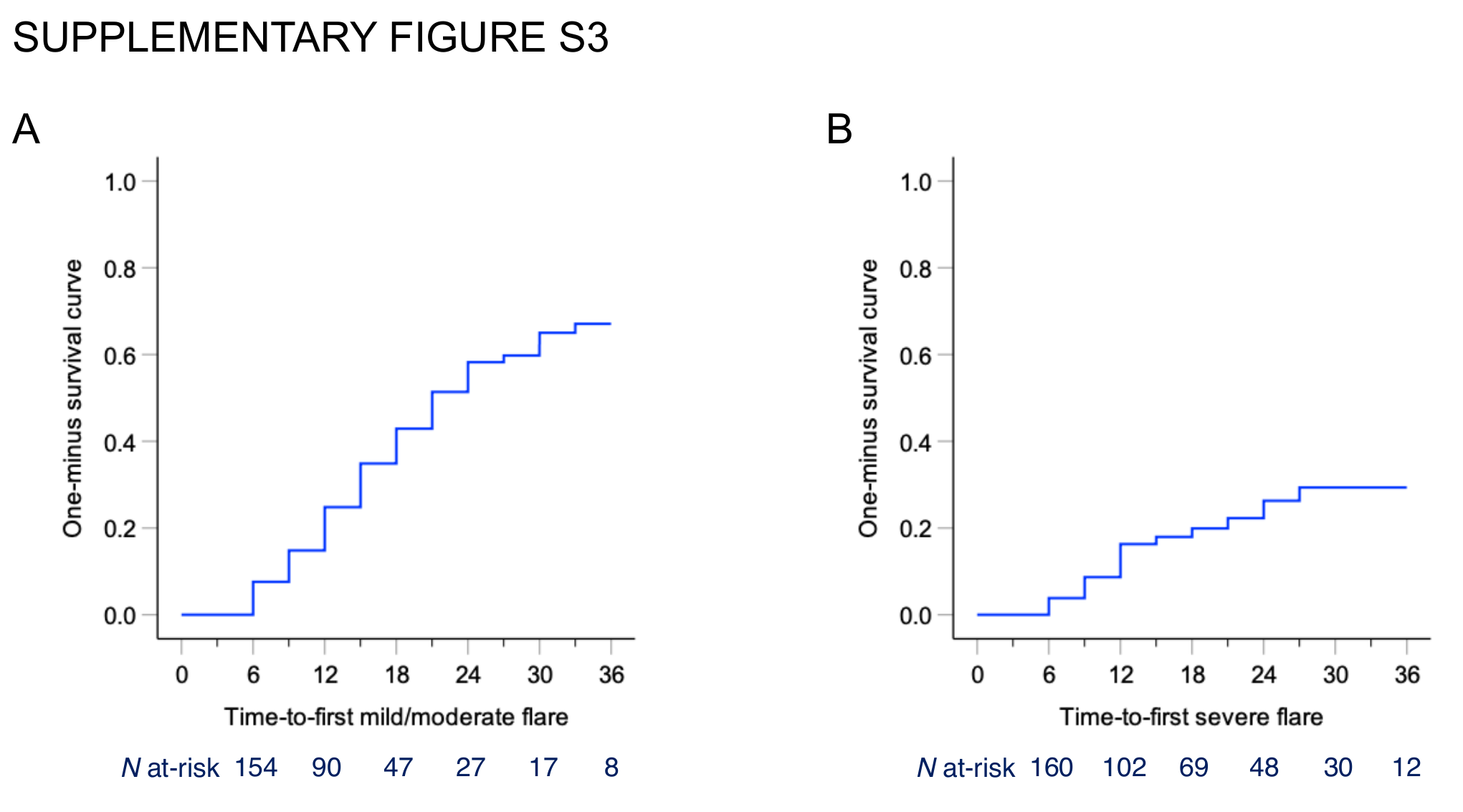

Supplement: Supplementary file 3 [file Image_3.tif]
