# Supplementary material for: Clinical response trajectories and drug persistence in systemic lupus erythematosus patients on belimumab treatment: A real-life, multicentre observational study
Source: Front Immunol. 2023 Jan 4;13:1074044. doi: 10.3389/fimmu.2022.1074044 (PMC9845912; doi:10.3389/fimmu.2022.1074044)
Supplement: Supplementary file 4 [file Table_1.docx]

**Supplementary Table S1.** Longitudinal trends of PGA, SLEDAI-2K, LLDAS and remission across the three trajectory-based groups (using data over 24 months) of response to belimumab

|  | **Trajectory-based groups of response to belimumab** (24 months) | | |
| --- | --- | --- | --- |
|  | **Complete response** ^1^ | **Partial response** ^2^ | **No response** ^3^ |
| **PGA** ^4^ |  | | |
| Baseline | 1.5 (0.5) ^5^ | 1.5 (0.5) | 2.0 (0.5) |
| Month 3 | 0.5 (0.5) | 1.0 (0.5) | 1.5 (1.0) |
| Month 6 | 0.0 (0.5) | 1.0 (0.6) | 1.5 (0.5) |
| Month 9 | 0.0 (0.5) | 1.0 (0.5) | 1.5 (0.5) |
| Month 12 | 0.0 (0.5) | 0.8 (0.5) | 1.3 (0.8) |
| Month 18 | 0.0 (0.6) | 0.5 (0.5) | 2.0 (1.0) |
| Month 24 | 0.1 (0.5) | 0.5 (0.6) | 0.9 (1.3) |
| **SLEDAI-2K** |  |  |  |
| Baseline | 7 (5) ^5^ | 8 (2) | 8 (4) |
| Month 3 | 2 (4) | 4 (2) | 6 (4) |
| Month 6 | 0 (4) | 4 (3) | 6 (4) |
| Month 9 | 0 (2) | 4 (4) | 6 (3) |
| Month 12 | 0 (2) | 4 (2) | 7 (5) |
| Month 18 | 1 (4) | 4 (2) | 8 (5) |
| Month 24 | 0 (4) | 4 (6) | 5 (5) |
| **LLDAS** ^6^ |  |  |  |
| Baseline | 6.3% | 0.0% | 0.0% |
| Month 3 | 68.2% | 36.0% | 10.9% |
| Month 6 | 81.4% | 37.5% | 7.7% |
| Month 9 | 60.0% | 51.0% | 5.0% |
| Month 12 | 67.7% | 52.1% | 12.5% |
| Month 18 | 63.2% | 55.2% | 5.3% |
| Month 24 | 76.5% | 35.3% | 33.3% |
| **Remission** ^7^ |  |  |  |
| Baseline | 0.0% | 0.0% | 0.0% |
| Month 3 | 14.9% | 1.3% | 0.0% |
| Month 6 | 46.5% | 2.9% | 0.0% |
| Month 9 | 43.3% | 5.1% | 0.0% |
| Month 12 | 41.4% | 7.7% | 0.0% |
| Month 18 | 36.8% | 8.6% | 0.0% |
| Month 24 | 50.0% | 15.0% | 0.0% |

^1^ N = 48, 47, 43, 36, 32, 21 and 20 for the time points month 0 (baseline), 3, 6, 9, 12, 18 and 24, respectively

^2^ N = 79, 76, 69, 59, 52, 34 and 22 for the time points month 0 (baseline), 3, 6, 9, 12, 18 and 24, respectively

^3^ N = 61, 55, 52, 41, 25, 19 and 6 for the time points month 0 (baseline), 3, 6, 9, 12, 18 and 24, respectively

^4^ Physician Global Assessment (scale 0-3)

^5^ Data are presented as median (interquartile range) values

^6^ Low Lupus Disease Activity State (defined in [30])

^7^ According to the DORIS 2021 definition.[31]

**Supplementary Table S2.** Longitudinal trends of PGA, SLEDAI-2K, LLDAS and remission across the three trajectory-based groups (using data over 12 months) of response to belimumab

|  | **Trajectory-based groups of response to belimumab** (12 months) | | |
| --- | --- | --- | --- |
|  | **Complete response** ^1^ | **Partial response** ^2^ | **No response** ^3^ |
| **PGA** ^4^ |  | | |
| Baseline | 1.5 (1.0) ^5^ | 1.5 (0.5) | 2.0 (0.5) |
| Month 3 | 0.5 (0.3) | 1.0 (0.5) | 1.8 (0.5) |
| Month 6 | 0.0 (0.5) | 1.0 (0.5) | 1.8 (0.5) |
| Month 9 | 0.0 (0.5) | 1.0 (0.8) | 2.0 (0.5) |
| Month 12 | 0.0 (0.5) | 1.0 (0.5) | 1.5 (1.0) |
| Month 18 | 0.5 (1.0) | 0.5 (0.5) | 1.3 (1.3) |
| Month 24 | 0.5 (1.0) | 0.5 (0.6) | 0.5 (0.8) |
| **SLEDAI-2K** |  |  |  |
| Baseline | 6 (4) ^5^ | 8 (2) | 8 (4) |
| Month 3 | 2 (3) | 4 (2) | 6 (3) |
| Month 6 | 0 (2) | 4 (2) | 6 (4) |
| Month 9 | 0 (2) | 4 (2) | 6 (3) |
| Month 12 | 0 (2) | 4 (4) | 6 (5) |
| Month 18 | 2 (4) | 4 (4) | 6 (6) |
| Month 24 | 0 (4) | 4 (5) | 2 (6) |
| **LLDAS** ^6^ |  |  |  |
| Baseline | 5.7% | 0.0% | 0.0% |
| Month 3 | 65.2% | 38.8% | 4.2% |
| Month 6 | 83.3% | 36.6% | 4.3% |
| Month 9 | 63.3% | 44.8% | 3.0% |
| Month 12 | 61.3% | 52.8% | 10.5% |
| Month 18 | 42.9% | 48.6% | 27.3% |
| Month 24 | 68.8% | 42.9% | 33.3% |
| **Remission** ^7^ |  |  |  |
| Baseline | 0.0% | 0.0% | 0.0% |
| Month 3 | 14.0% | 1.3% | 0.0% |
| Month 6 | 45.5% | 2.7% | 0.0% |
| Month 9 | 45.2% | 3.0% | 0.0% |
| Month 12 | 41.9% | 5.4% | 0.0% |
| Month 18 | 23.8% | 12.8% | 0.0% |
| Month 24 | 37.5% | 21.7% | 25.0% |

^1^ N = 53, 50, 45, 37, 34, 23 and 19 for the time points month 0 (baseline), 3, 6, 9, 12, 18 and 24, respectively

^2^ N = 84, 80, 73, 66, 56, 38 and 24 for the time points month 0 (baseline), 3, 6, 9, 12, 18 and 24, respectively

^3^ N = 51, 48, 46, 33, 19, 13 and 5 for the time points month 0 (baseline), 3, 6, 9, 12, 18 and 24, respectively

^4^ Physician Global Assessment (scale 0-3)

^5^ Data are presented as median (interquartile range) values

^6^ Low Lupus Disease Activity State (defined in [30])

^7^ According to the DORIS 2021 definition.[31]

**Supplementary Table S3.** Trajectory-based groups of response to belimumab in association with demographic and clinical features in patients with SLE

|  | **Trajectory-based groups of response to belimumab** | | | | |  |
| --- | --- | --- | --- | --- | --- | --- |
|  | **Complete response** | | **Partial**  **response** | | **No**  **response** | **P value**^1^ |
| Gender (female) | 97.9% | | 94.9% | | 95.1% | 0.688 |
| Age (years) | 52.0 (19.4) | | 47.9 (18.4) | | 46.5 (17.5) | 0.088 |
| Disease duration (years) | 8.9 (8.9) | | 5.5 (8.7) | | 6.4 (9.4) | 0.244 |
| Non smokers | 38.3% | | 56.2% | | 59.3% | 0.075 |
| Previous treatments | 2 (2) | | 2 (2) | | 2 (2) | 0.394 |
| HCQ ^2^ | 75.0% | | 79.7% | | 80.3% | 0.763 |
| Methotrexate | 68.8% | | 77.2% | | 70.5% | 0.511 |
| Leflunomide | 16.7% | | 21.5% | | 14.8% | 0.562 |
| Azathioprine | 60.4% | | 46.8% | | 59.0% | 0.218 |
| Mycophenolate | 12.5% | | 7.6% | | 23.0% | **0.032** |
| Cyclophosphamide | 16.7% | | 16.5% | | 19.7% | 0.869 |
| Rituximab | 12.5% | | 7.6% | | 13.1% | 0.512 |
| Other biologics | 2.1% | | 1.3% | | 3.3% | 0.715 |
| Concomitant use of HCQ | 65.5% | | 74.3% | | 77.6% | 0.425 |
| Concomitant use of ISTs ^3^ | 60.4% | | 73.4% | | 82.0% | **0.042** |
| Disease state |  | |  | |  |  |
| SLEDAI-2K | 7 (5) | | 8 (2) | | 8 (4) | **0.021** |
| Arthritis | 81.3% | | 92.4% | | 83.6% | 0.137 |
| Inflammatory rash | 31.3% | | 60.8% | | 70.5% | **<0.001** |
| Hair loss | 33.3% | | 35.4% | | 31.1% | 0.867 |
| Mucosal ulcers | 16.7% | | 24.1% | | 27.9% | 0.384 |
| Leukopenia | 14.6% | | 1.3% | | 13.1% | **0.010** |
| Thrombocytopenia | 2.1% | | 2.5% | | 4.9% | 0.641 |
| Fever | 6.3% | | 2.5% | | 1.6% | 0.361 |
| Pericarditis | 0.0% | | 2.5% | | 1.6% | 0.543 |
| Pleural effusion | 4.2% | | 0.0% | | 1.6% | 0.192 |
| Vasculitis | 2.1% | | 0.0% | | 3.3% | 0.293 |
| Hematuria | 2.1% | | 1.3% | | 4.9% | 0.198 |
| Proteinuria | 2.1% | | 1.3% | | 3.3% | 0.180 |
| Low complement | 37.5% | | 25.3% | | 41.0% | 0.119 |
| Anti-dsDNA | 12.5% | | 17.7% | | 26.2% | 0.178 |
| PGA ^4^ | 1.5 (0.5) | | 1.5 (0.5) | | 2.0 (0.5) | **<0.001** |
| Organ damage (SDI ^5^ >0) | 39.6% | | 39.7% | | 39.3% | 0.999 |
|  |  |  | |  | |  |

^1^ Chi-squared test or Kruskal-Wallis non-parametric test; unadjusted p-values are shown.

^2^ Hydroxychloroquine

^3^ Immunosuppressive treatment(s)

^4^ Physician Global Assessment

^5^ SLICC/ACR damage index

**Supplementary Table S4.** Adverse events resulting in discontinuation of belimumab

| **Description of adverse event** | **No. patients** |
| --- | --- |
| Bacterial infection | 5 |
| Viral infection | 1 |
| Opportunistic infection | 1 |
| Malignancy |  |
| Solid tumor | 2 |
| Hematological malignancy | 1 |
| Increased serum creatinine | 1 |
| Psychiatric manifestations | 4 |
| Rash – skin reaction | 2 |
| Infusion reaction | 1 |
| Menorrhagia | 1 |

**Supplementary Table S5.** Cox-regression analysis for the identification of factors associated with belimumab treatment discontinuation

|  | **Univariable analysis** | |  | **Multivariable analysis** | |
| --- | --- | --- | --- | --- | --- |
|  | HR (95% CI) ^1^ | P value |  | HR (95% CI) | P value |
| ***Efficacy-related discontinuation*** | |  |  |  |  |
| SLEDAI-2K (per 1-unit) | 1.11 (1.02–1.20) | 0.020 |  | – |  |
| PGA (per 1-unit) ^2^ | 2..82 (1.37–5.81) | 0.005 |  | 2..78 (1.32–5.85) | 0.007 |
| Rash (SLEDAI-2K) | 1.71 (0.99–2.93) | 0.053 |  | – |  |
| Pericarditis (SLEDAI-2K) | 3.21 (0.78–13.28) | 0.107 |  | – |  |
| Use of IST ^3^ | 1.85 (0.93–3.66) | 0.079 |  | – |  |
| ΔPGA^4^ by ≥0.5 (3 months) | 0.54 (0.32–0.92) | 0.023 |  | 0.57 (0.33–0.97) | 0.039 |
|  |  |  |  |  |  |
| ***Safety-related discontinuation*** | |  |  |  |  |
| Use of HCQ ^5^ | 0.33 (0.13–0.85) | 0.022 |  |  |  |

^1^ Hazard ratio (95% confidence interval)

^2^ Physician Global Assessment (scale 0-3)

^3^ Immunosuppressive treatment(s) (including methotrexate, azathioprine, mycophenolate)

^4^ Change (reduction) in PGA

^5^ Hydroxychloroquine
